# Zidovudine ameliorates pathology in the mouse model of Duchenne muscular dystrophy via P2RX7 purinoceptor antagonism

**DOI:** 10.1186/s40478-018-0530-4

**Published:** 2018-04-11

**Authors:** Rasha Al-Khalidi, Chiara Panicucci, Paul Cox, Natalia Chira, Justyna Róg, Christopher N. J. Young, Rhiannon E. McGeehan, Kameshwari Ambati, Jayakrishna Ambati, Krzysztof Zabłocki, Elisabetta Gazzerro, Stephen Arkle, Claudio Bruno, Dariusz C. Górecki

**Affiliations:** 1 Molecular Medicine Laboratory, School of Pharmacy and Biomedical Sciences, University of Portsmouth, Portsmouth, UK; 2 Center of Myology and Neurodegenerative Disorders, Department of Neuroscience and Rehabilitation, Istituto Giannina Gaslini, Genoa, Italy; 3 Laboratory of Cellular Metabolism, Department of Biochemistry, Nencki Institute of Experimental Biology of the Polish Academy of Sciences, Warsaw, Poland; 4 Department of Ophthalmology & Center for Advanced Vision Science, University of Virginia, Charlottesville, VA USA; 5 Department of Pathology, Microbiology, Immunology & Cancer Biology, University of Virginia, Charlottesville, VA USA; 6 Present Address: Biotechnology Department, Faculty of Science, University of Baghdad, Baghdad, Iraq; 7 Present Address: School of Allied Health Sciences, Faculty of Health and Life Sciences, De Montfort University, Leicester, UK

**Keywords:** AZT, Duchenne muscular dystrophy, eATP, mdx, Purinergic receptors, P2RX7

## Abstract

Duchenne muscular dystrophy (DMD) is the most common inherited muscle disorder that causes severe disability and death of young men. This disease is characterized by progressive muscle degeneration aggravated by sterile inflammation and is also associated with cognitive impairment and low bone density. Given that no current treatment can improve the long-term outcome, approaches with a strong translational potential are urgently needed. Duchenne muscular dystrophy (DMD) alters P2RX7 signaling in both muscle and inflammatory cells and inhibition of this receptor resulted in a significant attenuation of muscle and non-muscle symptoms in DMD^mdx^ mouse model. As P2RX7 is an attractive target in a range of human diseases, specific antagonists have been developed. Yet, these will require lengthy safety testing in the pediatric population of Duchenne muscular dystrophy (DMD) patients. In contrast, Nucleoside Reverse Transcriptase Inhibitors (NRTIs) can act as P2RX7 antagonists and are drugs with an established safety record, including in children. We demonstrate here that AZT (Zidovudine) inhibits P2RX7 functions acting via the same allosteric site as other antagonists. Moreover, short-term AZT treatment at the peak of disease in DMD^mdx^ mice attenuated the phenotype without any detectable side effects. Recovery was evident in the key parameters such as reduced sarcolemma permeability confirmed by lower serum creatine kinase levels and IgG influx into myofibres, decreased inflammatory cell numbers and inflammation markers in leg and heart muscles of treated mice. Moreover, this short-term therapy had some positive impact on muscle strength in vivo and no detrimental effect on mitochondria, which is the main side-effect of Nucleoside Reverse Transcriptase Inhibitors (NRTIs). Given these results, we postulate that AZT could be quickly re-purposed for the treatment of this highly debilitating and lethal disease. This approach is not constrained by causative DMD mutations and may be effective in alleviating both muscle and non-muscle abnormalities.

**Electronic supplementary material:**

The online version of this article (10.1186/s40478-018-0530-4) contains supplementary material, which is available to authorized users.

## Introduction

Duchenne muscular dystrophy (DMD) is the most common inherited muscle disorder with X-linked inheritance. Affected boys suffer from a progressive muscle degeneration and weakness, which lead to loss of ambulation in early teens. Skeletal deformities, respiratory insufficiency and cardiomyopathy developing in the second decade of life are typical for this debilitating and still lethal disease. The pleiotropic effects of the mutant gene also include non-muscle symptoms: cognitive impairment and structurally weakened bones [3, 55]. DMD is caused by mutations in the DMD gene encoding a range of dystrophin proteins. The muscle isoform is a 427 kDa protein with a role in anchoring the dystrophin-associated protein complex (DAPC) in the muscle sarcolemma. Lack of dystrophin is attributed to plasma membrane destabilization, cell signaling impairment and myofibre necrosis, accompanied by chronic sterile inflammation, and finally leading to irreversible replacement of muscle with fibrotic and adipose tissues [52]. Most recent studies indicated that expression of specific dystrophin isoforms is also important for the proper functioning of myogenic cells [19, 72] and dystrophinopathy is also responsible for the cognitive impairment and bone weakness [3, 41, 55, 59].

Importantly, numerous studies demonstrated that chronic muscle inflammation plays a crucial role in DMD pathogenesis. Specifically, in the *mdx* mouse model of DMD, treatments inducing depletion of CD4, CD8, neutrophils or macrophages or of anti-cytokine therapies significantly improved the dystrophic phenotype [13, 20, 28, 45, 60]. The mechanism underlying the sterile inflammation in dystrophic muscle is not completely understood but damage-associated molecular patterns (DAMPs) released from damaged myofibres seem to be the key factor. ATP released into the extracellular space (eATP) is one of the most important DAMPs acting as a “danger signal” triggering inflammation via activation of the P2RX7 purinoceptors. This “danger receptor” belongs to a family of ATP-gated ion channels. However, unlike other P2RXs, it requires millimolar concentrations of eATP for full activation [30]. Such high eATP levels are only encountered in pathologies. P2RX7 triggers complex downstream signaling producing increased IL-1b levels and the NLRP3 inflammasome activation. Interestingly, P2RX7 expression and activation in inflammatory cells has been well documented [48] but recent studies also showed a significant up-regulation of this purinoceptor in muscle cells from the mouse model of DMD [8, 54, 73]. When exposed to eATP, dystrophic DMD^*mdx*^ myoblasts respond with increased cytosolic Ca^2+^ influx and IL-1b release, suggesting that skeletal muscle cells can actively participate in the inflammatory process through purinergic signaling [54]. Moreover, high eATP acting on P2RX7 activates both abnormal Ca^2+^ influx and large pore opening triggering a unique mechanism of autophagic cell death [75] and increased MMP-2 activation [74]. Treatment with apyrase, an ATP degrading enzyme, reduced intracellular Ca^2+^ levels in *mdx* fibers [2] and P2RX7 antagonists reduced the cell death and MMP-2 activity [74, 75], thus confirming that P2RX7 contributes to the deregulated homeostasis in dystrophic muscles.

Therefore, activation of P2RX7 pathways in DMD results in direct muscle cell damage and death as well as an enhanced inflammatory response worsening the muscle pathology in a mechanism akin to the involvement of P2RX7 in other inflammatory diseases [14, 17].

We have previously demonstrated the therapeutic impact of both genetic ablation and pharmacological blockade of P2RX7 in *mdx* mice in vivo. This included significant improvements in muscle morphology and strength but also a significant reduction of the inflammatory phenotype [24, 58] as well as amelioration of non-muscle symptoms [41, 58]. This wide range of improvements reflects the involvement of P2RX7 in multiple disease mechanisms. Therefore, P2RX7 blockade emerges as an attractive target for translational approaches. Numerous P2RX7 antagonists have been developed [9, 27, 37, 63] and some of these e.g. AZD9056 and CE-224,535, have been used in clinical trials in inflammatory diseases [21, 34, 62]. However, none of these compounds have been approved as medicines and none tested in children.

Importantly, Fowler et al., [22] demonstrated that the Nucleoside Reverse Transcriptase Inhibitor (NRTI) class of compounds, commonly used as anti-HIV drugs, can act as P2RX7 antagonists [22, 40]. These drugs, with established safety records, could be re-purposed for the treatment of this lethal disease. However, the mode of action of NRTI at the receptor and their efficacy in the specific pathology such as DMD were unknown.

Here we used a molecular modelling approach to establish whether NRTIs act as classical P2RX7 antagonists. Furthermore, we tested Zidovudnine (Azidothymidine, AZT), one of the mainstay NRTI therapeutics in HIV prevention and treatment, for its anti-P2RX7 properties in dystrophic muscle cells in vitro and for its efficacy in alleviating the pathology in the mouse model of DMD in vivo. It is the consensus opinion of experts that the *mdx* mouse is currently the most appropriate pre-clinical model to test treatment efficacy for DMD (http://www.treat-nmd.eu/research/preclinical/dmd-sops/). We established that even a short-term treatment with AZT significantly reduced muscle inflammation and improved the dystrophic phenotype. Consequently, AZT, which has established pharmacological profile also in the pediatric population, is an ideal candidate for rapid re-purposing as a DMD therapeutic.

## Materials and methods

### Molecular modelling

Molecular docking of the NRTIs: AZT, 2-Me-AZT [44], d4T and me-d4T in the P2RX7 receptor was performed using the Molecular Operating Environment (MOE) program [42]. The crystal structure for the giant panda P2RX7 with the ligand A804598 bound (5U1V) was downloaded from the Protein Database [5]. Human and mouse P2RX7 peptide sequences from UniProt were aligned with the Giant panda (Additional file 1: Figure S1) to establish the sequence homology of the critically important regions. The Protonate3D tool was used to add hydrogen atoms to the protein and partial charges were calculated for the protein atoms. Ligand binding sites were generated using the MOE Alpha Site Finder tool, which generates hydrophobic and hydrophilic α-spheres that are clustered together to represent regions of empty space in the protein. Ligand molecules were constructed using the builder tool, partial charges were calculated and the ligand molecule was energy minimized using the MMFF94× (Merck Molecular Forcefield 94×) prior to commencing a docking run. The Docking module was used to dock the ligand into appropriate ligand binding sites using ‘Triangle Matcher’ placement methodology and 300 placement poses. The lowest energy 100 unique receptor-ligand complexes were identified and scored according to the London dG scoring function. They were then submitted to a final forcefield minimization step retaining 100 unique ligand receptor complexes. The docking score, S, was calculated using the Generalised Born Solvation model (GB/VI). This interaction is the non-bonded interaction energy (van der Waals, Coulomb and GB implicit solvent interaction energies) between the receptor and the ligand. The self-energies of both the receptor and the ligand are excluded. Energy minimization was carried out using the conjugate gradient method with a cutoff distance of 6 Å and a convergence criterion of 0.04 kJ/mol. The atoms of the receptor were held fixed during the calculations.

### Animals

All animal experiments were performed in accordance with the Principles of Laboratory Animal Care (NIH publication No. 86–23, revised 1985) and approvals of the Institutional Ethical Review Board and the Home Office UK (70/7479). Our experimental designs exploit the ARRIVE guidelines [58] and the TREAT-NMD guidelines designed to standardize experimental protocols used as efficacy readouts to allow comparisons of parallel efforts. Starting at 4 weeks of age, *mdx* male mice (unbiased by gender) were treated by i.p. injection twice a day either for 2 or 4 weeks with 50 mg/kg body weight of AZT (Zidovudine; Z1900000) or for 2 weeks with 25 mg/kg body weight of 2-Me-AZT. Dosage was based on previous studies [12, 22, 40]. Age-matched control mice received the same volume of phosphate buffered saline (pH 7.4, filter-sterilized, Sigma Aldrich).

Following the treatment, mice were killed by carbon dioxide inhalation; serum, tibialis anterior (TA), gastrocnemii (GC), and heart muscles were collected for further evaluations. Investigators were blinded to the sample group allocation, where possible.

### Cell culture

Dystrophic *mdx* myoblasts [75] were cultured as primary cells in KnockOut DMEM (Invitrogen) supplemented with 10% ^*v*^/_v_ KSR (Knockout Serum Replacement, Invitrogen), 5% ^v^/_v_ DHS (Donor Horse Serum, Sera Labs) and 2 mM L-glutamine.

### The P2RX7 large pore assay

For dye uptake assays, cells cultured under conditions described above were washed and incubated in the large pore buffer (145 mM NaCl, 5 mM KCl, 1 mM MgCl_2_ and 10 mM Na-HEPES, pH 7.4) containing 5 μM EtBr. Following addition of agonists with or without AZT or me-AZT, EtBr uptake was analyzed under LSM510 confocal microscope (Zeiss) with heated stage at 37 °C and a dipping objective, or using a POLARstar Optima plate reader (BMG Labtech). Each experiment was repeated at least 3 times.

### Intracellular Ca2+ measurements

Myoblasts were cultured on glass coverslips in a 6-well plate (500,000 cells/well) for 48 h under conditions described above. Cells (70–80% confluent) were loaded with Fura-2 AM (Molecular Probes, Oregon) in culture medium for 15 min at 37 °C in a 95% O_2_, 5% CO_2_ atmosphere. After 2 brief washes in the assay buffer (130 mM NaCl, 5 mM KCl, 2 mM CaCl_2_, 1 mM MgCl_2_, 0.5 mM Na_2_HPO_4_, 1 mM pyruvate, 5 mM D-glucose, and 25 mM HEPES; pH 7.4), the coverslips were mounted in a cuvette and maintained in the assay buffer at room temperature in an RF5301PC spectrofluorimeter (Shimadzu). Fluorescence was recorded at 510 nm with excitation at 340/380 nm. At the end of each experiment the Fura 2 fluorescence was calibrated by addition of 13 μM ionomycin to determine maximal fluorescence ratio followed by addition of EGTA to complete removal of Ca^2+^. Cytosolic Ca^2+^ concentration [Ca^2+^]c was calculated according to Grynkiewicz et al. [26]. The cells were treated with: 300 μM BzATP; 20 μM AZT and 20 μM me-AZT (applied 30 and 10 min prior to the addition of BzATP, respectively). Each experiment was repeated at least 4 times for AZT and 3 times for me-AZT.

### Serum CK level measurement

The blood samples were collected, allowed to coagulate and centrifuged for 10 min at 2500 g. Immediately after centrifugation, the serum was isolated and stored at -20 °C. The creatine kinase (CK) levels were analyzed using the Creatine Kinase Activity Assay Kit (Mak116-1kt, Sigma-Aldrich), according to manufacturer’s instructions.

### Treadmill test

A five lane treadmill (Panlab/Harvard Apparatus) equipped with a darkened far-end area to encourage running, was used. AZT- and PBS-treated *mdx* mice were challenged on the treadmill twice (at the end of the second and the fourth week of treatment) for 30 min according to the protocol described by Radley-Crabb [51]. Briefly, groups of 4 mice were settled for 2 min on the treadmill with a stationary belt, then acclimatized for 2 min at a speed of 4 m/min, warmed up for 8 min at 8 m/min and finally exercised for 30 min at 12 m/min. The number of stops during the last 30 min and the total period spent running for each animal were measured.

### Forelimb grip strength test

AZT- and PBS-treated *mdx* mice were also compared in the forelimb grip strength test (Force Gauce FG-5000A, Lutron Electronic) at the end of the second and the fourth week of treatment according to the SOP (ID) DMD_M.2.2.001. Briefly, each trial consisted of five repetitions with at least 1 min elapsing between each of the five determinations per animal; the grip strength value for each mouse was recorded as the average of the three best efforts and was then divided by the mouse body weight.

### Histochemical analysis

Sections (10 μm) from frozen tibialis anterior and heart muscles isolated from 8 weeks old AZT- and PBS-treated *mdx* mice were cut on a cryostat. Sections were obtained from the middle third of the muscle, collected on poly-L-lysine (0.5 mg/ml) – coated glass slides and subsequently stained with H&E and/or acid phosphatase (AP). AP staining was used to quantify the inflammatory infiltrate areas, exploiting the properties of AP-rich inflammatory cells producing an azo dye when coupled with a naphthol-based buffer. For AP staining frozen muscle sections were kept at ambient temperature for 30′. Afterwards sections were incubated for 1 h at 37 °C with the incubating solution made as follow: substrate solution (naphtol AS-B1 phosphate 0.02 M in dimethylformamide), buffer solution (veronal acetate 0.15 M), sodium nitrite 4% (*w*/*v*) and pararosaniline solution (pararosaniline 0.12 M in 2 N HCl). Sections were then dehydrated in ascending alcohols (50%, 70%, 80%, 95% X2, 100% X2), cleared with xylene and mounted with Permount [4]. The AP-positive (red signal) areas were captured using an automated method through the Ariol system (Leica Biosystem) for an unbiased analysis and expressed as % of the total cross-sectional area.

### Immunofluorescence

10 μm thick cryosections were fixed in a 4% ^*w*^/_*v*_ paraformaldehyde solution in TBST for 15 min at 4 °C. The primary antibody incubation in TBST containing 10% ^*v*^/_v_ serum was applied overnight at 4 °C and secondary antibody incubation in TBST and 2% ^v^/_v_ serum containing Hoechst fluorescent nuclear counterstain was applied for 1 h at room temperature. Sections were mounted using Fluor Preserve Reagent (Merk Millipore) mounting medium.

The following antibodies were used: CD68—MCA1957GA rat monoclonal (AbD Serotec), dilution 1:500; collagen type-IV—AB769 goat polyclonal (Chemicon), dilution 1:500; Dystrophin- mouse monoclonal (D8043 SIGMA Sigma Aldrich), dilution 1:500; Ly6G—14–593 rat monoclonal (eBioscience), dilution 1:250; P2RX4 rabbit polyclonal (Alomone), dilution 1:500. Images were captured using a confocal microscope (LSM 710, Zeiss); the whole cross-section area from TA or GC was captured by non-overlapping 10× magnification images.

Muscle fiber size and central nucleation were visualized by collagen type-IV and Hoechst immunofluorescence staining. Individual microscope fields-of-view were montaged using ImageJ to present whole cross-sections through the muscle. Image analysis was performed on these composite images using Fiji, ImageJ, open-source software (NIH, US). A macro developed as described [58] was used to measure morphometric variables including the minimum Feret diameter, the total fiber number and the centrally nucleated fiber number per analyzed area.

For P2RX4, CD68 and Ly6G detection, the whole TA section was scanned with the confocal microscope at 10× magnification and quantification of immunofluorescent signals was performed with a semi-automated (unbiased) method using a thresholding macro in ImageJ and divided by the number of fields thus obtaining a number expressed in arbitrary units (AU), which indicates the mean fluorescence signal per unit area. The same method was applied to quantify IgG permeability into muscle fibers. Dystrophin staining to identify the revertant fibers was performed in TA muscle sections. Hoechst nuclear counterstain was used to help visualizing the total number of myofibers present in each section. Revertant fibers were manually enumerated and reported as percentage of total myofibers.

### Western blotting

Total proteins from frozen TA muscles were extracted by crushing samples with a mortar and pestle under liquid nitrogen and further homogenization in the extraction buffer: 100 mg of muscle powder were homogenized in 500 μl of complete lysis M reagent (Sigma-Aldrich) enriched with protease inhibitor cocktail 1X and phosphatase inhibitor cocktail 2X (all Roche). All samples were centrifuged at 800 g for 3 min at 4 °C, and protein concentrations were determined using a Bicinchoninic Acid Kit (Sigma-Aldrich). 50 μg of protein was mixed at 1:1 ^*v*^/_v_ ratio with Laemmli buffer 2X with 5% ^v^/_v_ β-mercaptoethanol, heated for 5 min at 95 °C and chilled on ice. Proteins were then resolved on 6%–12% ^*w*^/_*v*_ SDS-polyacrylamide gels and transferred onto Hybond PVDF membranes (Amersham). Membranes were blocked in 5% ^w^/_v_ non-fat milk powder in 1× TBST with 0.01% ^v^/_v_ Tween 20 (Sigma-Aldrich) for 1 h, then probed overnight at 4 °C with primary antibody diluted in the same blocking buffer, and finally incubated with the appropriate horseradish-peroxidase-conjugated secondary antibody (Sigma-Aldrich) for 1 h at room temperature. Specific protein bands were visualized using Laminata Western HRP Substre (Millipore) and images were obtained using a ChemiDoc MP system (BioRad). All densitometric analyses of protein bands were made using exposure times within the linear range and the integrated density measurement function of the imaging software. The CD11b antibody, clone M1/70 rabbit (Abcam) was used at a 1:1000 dilution and GAPDH (Santa Cruz) goat polyclonal antibody was used at 1:500 and served as the protein-loading control.

### RT-qPCR

Total RNA was extracted from muscles using RNeasy Mini Kit (Qiagen) according to the manufacturer’s protocol. Total RNA, containing miRNA was also extracted from sera of AZT- and PBS-treated *mdx* mice after 2 weeks of treatment according to the manufacturer’s protocol for the miRNeasy Serum/Plasma kit (Qiagen). Quality and quantity was assessed using a NanoDrop spectrophotometer.

1 μg of RNA was reverse transcribed using a Super-ScriptTM VILO cDNA Synthesis Kit (Invitrogen). For the RT-qPCR amplification, 25 ng and 12.5 ng of cDNA (respectively for the target genes and for GAPDH control) were used in 20 μl reaction volume prepared with TaqMan Universal Master MIX II (Applied Biosystem) or SYBR Green PrecisionPLUS qPCR MasterMix (Primer Design). Each sample was run in duplicate using a ViiA7 Real Time PCR Detection System (Applied Biosystems, USA). The expression of target genes relative to GAPDH was determined by using the △CT method [57] The primers used were as follows:

Taqman probe NCBI accession numbers: CD68: NM_001291058.1, CD163: NM_001170395.1, P2X4: NM_011026, CD4: NM_013488.2, CD8a: NM_001081110.2, Foxp3: NM_001199347.1, LY6G: NM_023463.3, TNF-a: NM_001278601.1, IL6: NM_031168.1, IL 10: NM_010548.2, IL-12a: NM_001159424.1, COX2: NC_005089.1, Bmp7: NM_007557.3, Mir206: NR_029593.1, Mfn2: NM_001285921.1, GAPDH: NM_008084.2.

SybrGreen primer sequences:

- TGFb: Fwd 5’-CTCCACCTGCAAGACCAT-3′; Rev 5’-CTTAGTTTGGACAGGATCTGG-3’
- IL33: Fwd TCCTTGCTTGGCAGTATCCA, Rev TGCTCAATGTGTCAACAGACG
- iNOS: Fwd CAGTTCCGAGCGTCAAAGACCTGC-3′, Rev CAGCCCAACAATACAATACAAGATG.
- IL1b: Fwd TCTGATGGGCAACCACTTAC, Rev GTTGACAGCTAGGTTCTGTTCT
- Nlrp3: Fwd TGAATCGGAACAACCTGAC, Rev CCACCAGCAAGAAGAAGC
- NF-kb: Fwd ACACGAGGCTACAACTCTGC, Rev GGTACCCCCAGAGACCTCAT

### MtDNA copy number analysis

The qPCR (absolute quantification) was performed on total DNA isolated from snap-frozen GC muscle isolated from AZT- and PBS-treated *mdx* mice after 4 weeks of treatment tissue and externally generated standards using Sybr green (BioRad) and primers specific for mitochondrial DNA (mtDNA): Fwd CAGTCTAATGCTTACTCAGC, Rev GGGCAGTTACGATAACATTG and GAPDH: FwD TCAAGCTCATTTCCTGGTATGAC, Rev CTTGCTCAGTGTCCTTGCTG. As two copies of GAPDH are present in every nucleus, GAPDH amplification data were divided by 2 to calculate the number of nuclei present in each sample. The number of mtDNA copies was then calculated by dividing the mtDNA amplification data by the number of nuclei [7, 49]. Measurements were made in duplicate. The analysis was carried out on 4 mice per experiment.

### Statistical analysis

For statistical analysis of cell assays a one-way analysis of variance (ANOVA) was performed with the post- hoc Tukey’s test (Microcal Origin 7.0). Results are reported as mean (+/-SD), where n refers to number of independent samples or individuals. Mann Whitney test was used for comparisons between the two data sets (PBS*-mdx* vs AZT-*mdx*). Two way- ANOVA with Bonferroni multiple comparisons were used to compare the PBS and AZT treatment in 2 and 4 weeks. For RTqPCR data sstatistical analysis was performed on the relative expression values with the Mann Whitney test and represented as Log2 fold change versus the mean PBS-*mdx*.

A *p*-value of < 0.05 was considered statistically significant, and the values are reported as follows in figures: *p < 0.05, **p < 0.01, ***p < 0.001.

## Results

It has not been known whether NRTIs bind directly to P2RX7 and, if so, where or whether they have an indirect effect. To gain insights into these questions, we have used molecular modeling and the recently published mammalian P2RX7 crystal structure [31] to identify putative binding sites for AZT (Zidovudine), to the P2RX7 molecule. AZT is one of the most widely used NRTIs. Moreover, the mechanism of action of NRTIs at the P2RX7 is independent from the reverse transcriptase inhibition because their methyl derivatives retain the P2RX7 antagonism but not the NRTI activity [22]. Therefore, we used the same approach to compare the most likely binding sites for 2-Me-AZT as well as for d4T and its 5’-O-methyl-modified derivative me-d4T.

### Modelling of AZT and its derivatives on P2RX7

Human and mouse P2RX7 peptide sequences from UniProt were aligned with the giant panda (Additional file 1: Figure S1) and showed 77% and 81% of residues to be entirely conserved between panda and mouse and human, respectively. Moreover, the key residues involved in binding specific P2RX7 antagonists (e.g. A804598) are also conserved, demonstrating that the panda structure represents the best current template for investigating the potential mode of action of AZT both in the mouse model and patients.

**Fig.1:**
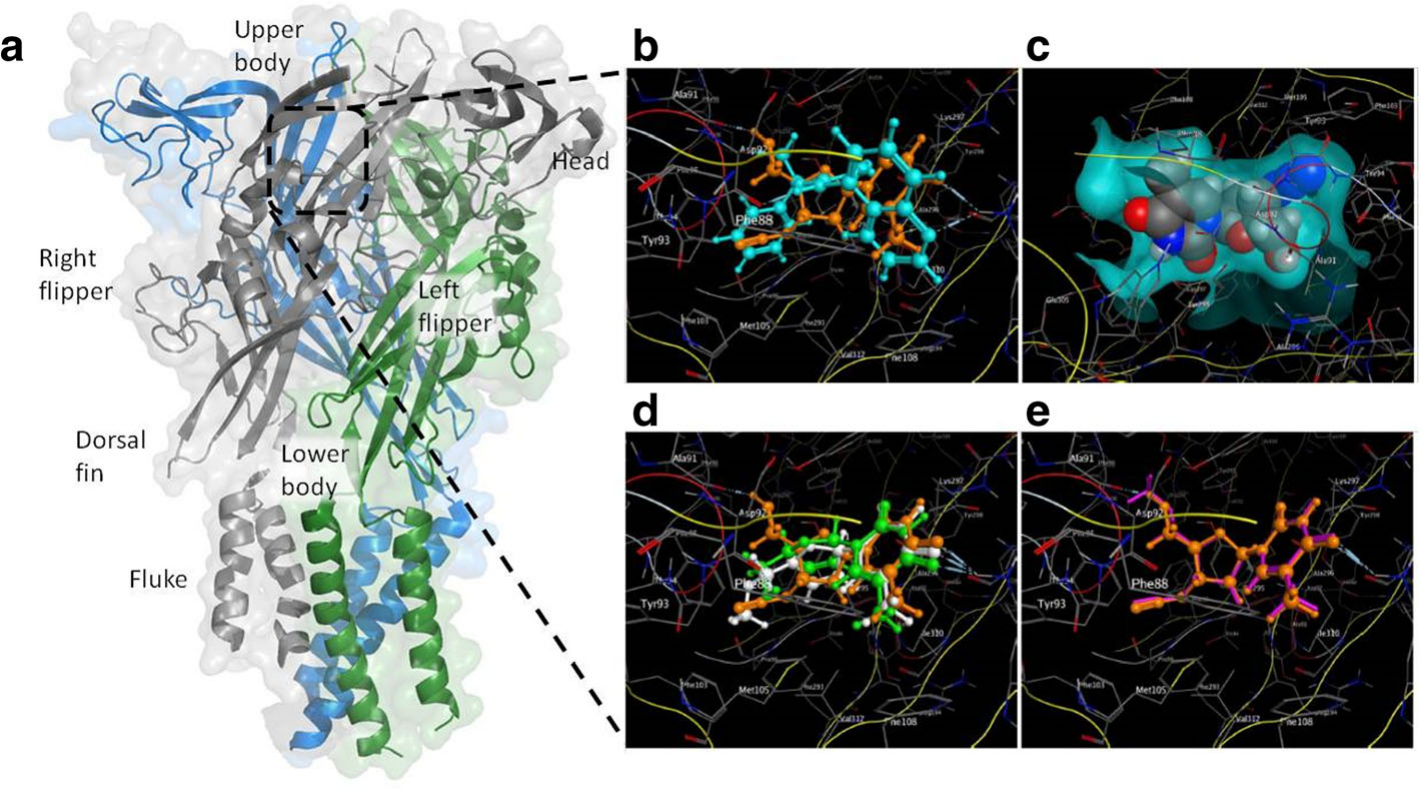
AZT binds to the same allosteric site as canonical P2RX7 antagonists. **a** Surface representation of trimeric P2X7, combining the Giant panda [PDB:5U1V] shown in grey and two chicken monomers [PDB:5XW6] shown (for illustration only) in blue and green. The approximate location of the allosteric site formed by a groove between two of the adjacent monomers is highlighted by the black box. **b** The optimized location of AZT (orange) compared with the experimental location of A804598 (cyan). Both molecules occupy very similar regions of space. **c** The optimized location of the AZT molecule inside the allosteric pocket (light blue). **d** The optimized locations of AZT (orange), d4T (green) and d4T-me (white). **e** The optimized locations of AZT (orange) and me-AZT (magenta). H-bond interactions are shown via a dotted line

The AZT molecule was docked into all of the 29 unique docking sites identified by the Alpha Site Finder Tool in MOE. The optimized binding energies for the AZT at all these sites are summarized in the Additional file 2: Table S1. The lowest energy position for AZT (− 89.3 kJ mol^− 1^) was found to be at the allosteric binding site identified by Karasawa and Kawate [31]. This is the position that several structurally distinct antagonists (A74003, A804598, AZ10606120, GW791343 and JNJ47965567) were found to occupy inside the P2X7 receptor (Fig. 1a). A comparison between the predicted location for AZT and the experimental location for A804598 is shown in Fig. 1b. It is clear that the AZT molecule is predicted to occupy the same region of space as A804598 and it fills the available space inside the allosteric binding site well (Fig. 1c).

The most favorable binding sites for both d4T and me-d4T were also found to be at the same allosteric site. Their optimized energies were − 74.7 and − 80.2 kJ mol^− 1^ respectively, very similar to the value obtained for AZT. The potentially enhanced binding affinity of the methylated molecule to the P2RX7 is likely to be due to enhanced van der Waals’ interaction between the ligand and the protein. The optimized locations of d4T, me-d4T and AZT are shown in Fig. 1d.

Docking 2-Me-AZT inside the receptor also finds the most favorable binding site to be at the allosteric one. The optimized energy is − 87.4 kJ mol^− 1^. Although the addition of the methyl group means that a hydrogen bond interaction with the residue Ala91 is lost, this is compensated by an increase in the van der Waals’ interaction, which results in a very similar binding energy to the one obtained for AZT. The optimized locations for these molecules are shown in Fig. 1e.

### AZT inhibits both the P2RX7 ion channel function and large pore formation in mdx myoblasts

**Fig. 2:**
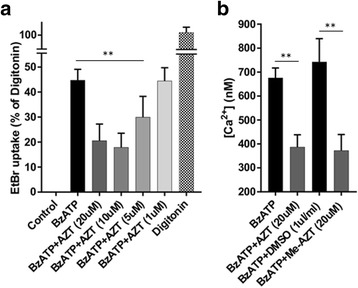
AZT inhibits P2RX7 large pore and channel functions in *mdx* myoblasts **a** EtBr uptake (expressed as % permeabilization evoked by 50 μg/ml digitonin) following 30 min exposure to 1 mM BzATP with and without the specified concentrations of AZT. Note the concentration-dependent inhibition up to 20 μM of AZT. One-way ANOVA, df = 5, *n* = 6, *p* = 0.008. **b** 1 mM BzATP-stimulated Ca^2+^ influx was inhibited in the presence of 20 μM AZT or me-AZT. One-way ANOVA, df = 3, *n* = 4, *p* = 0.001. Error bars show mean ± SD, **p < 0 .01

The modelling data clearly points to AZT and 2-Me-AZT binding to the P2RX7 allosteric site. We tested whether these drugs inhibit this receptor in dystrophic muscle cells. P2RX7 functions as a Ca^2+^ permeable ion channel and, upon prolonged ATP stimulation, as a large pore. We have previously shown that A74003 can inhibit both of these functions in *mdx* cells [73, 75]. Pre-incubation of dystrophic myoblasts with AZT at 1, 5, 10 and 20 μM resulted in a concentration-dependent inhibition of the large pore opening measured by the EtBr uptake (Fig. 2a). While 1 μM AZT had no effect on channel or pore functions the inhibitory effect appeared to reach saturation at around 10–20 μM and, at these concentrations, AZT also blocked the Ca^2+^ influx (Fig. 2b). Furthermore, 20 μM me-AZT had the same inhibitory effect on the Ca^2+^ influx as equimolar AZT. Importantly, the ~ 50% inhibition evoked by AZT was comparable to the effects obtained previously using other antagonists [75]. Additionally, cells incubated with AZT or 2-Me-AZT were observed to retain a greater degree of elongation and adherence compared to cells exposed to the specific P2RX7 antagonist (data not shown), indicating that these drugs are better tolerated by muscle cells at inhibitory concentrations.

### AZT treatment reduces skeletal and heart muscle inflammation in mdx mice in vivo

**Fig. 3:**
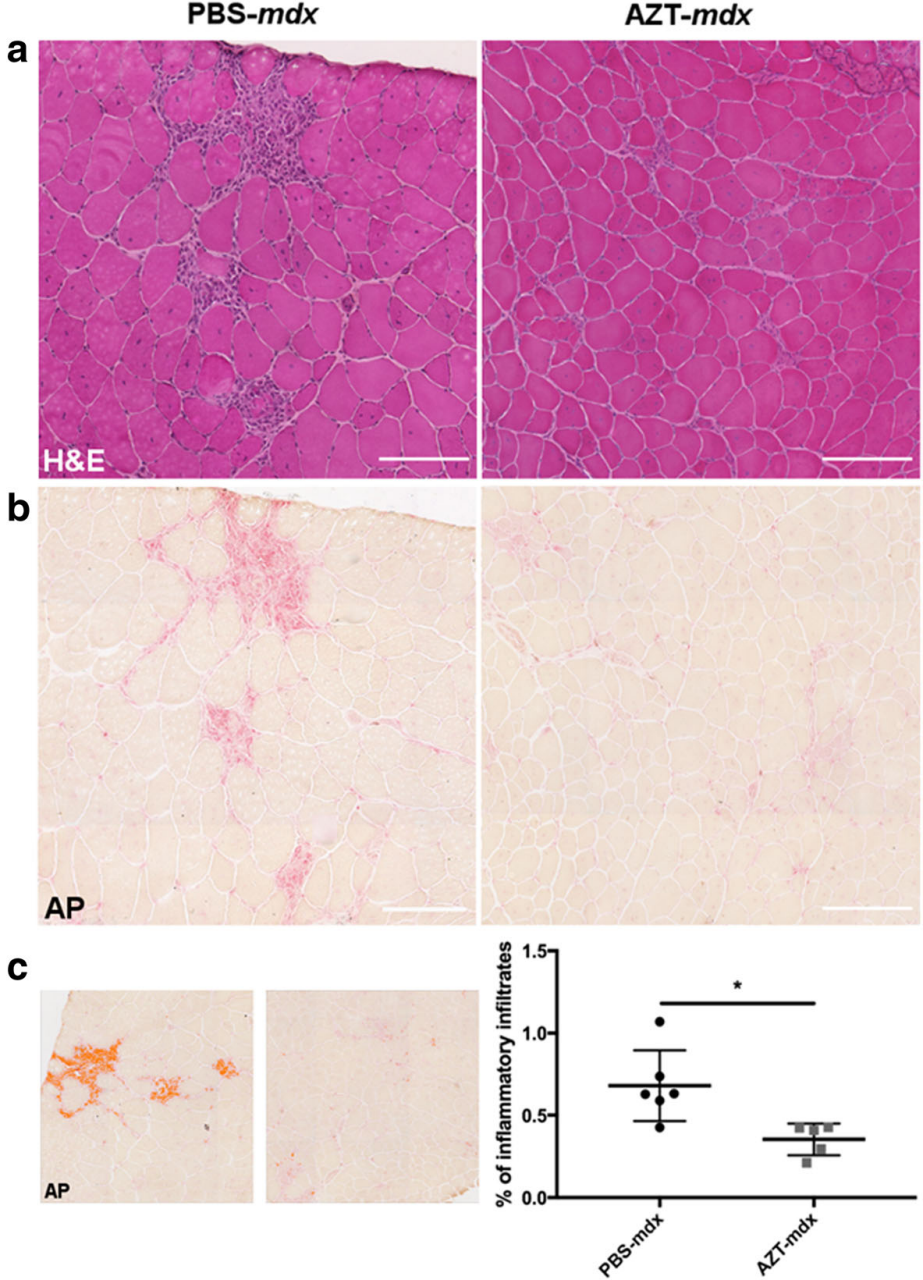
AZT decreases inflammatory infiltrates in *mdx* muscle. Representative images of TA sections following 4 week treatment with PBS or AZT, stained with H&E (**a**) and AP (**b**). AZT-*mdx* shows smaller inflammatory infiltrate areas than PBS-*mdx.* Scale bar = 100 μm. The AP signal indicating inflammatory cells (**c**), was quantified using the automated image analysis system (Ariol) with the AP positive area calculated as % of the total cross-sectional area. Mann Whitney test, *n* = 6, *p* = 0.012. *p < 0.05

To mimic the clinically relevant situation, treatment and analyses were performed at the age when *mdx* mice show the typical muscle degeneration and regeneration and the inflammatory cell infiltration pattern akin to human pathology. The 4 week old *mdx* males were injected i.p. with 50 mg/kg body weight AZT twice a day for 2 or 4 weeks. The weight gain profiles of mice treated with AZT showed no difference to the PBS-treated controls (Additional file 3: Figure S2). Inflammatory muscle infiltrates were evaluated in tibialis anterior (TA) muscles isolated from *mdx* mice injected with AZT or PBS for 4 weeks. H&E (Fig. 3a) and acid phosphatase (AP) staining (Fig. 3b) showed that AZT treatment significantly decreased the inflammatory infiltrations surrounding the necrotic muscle fibers. The morphometric AP analysis (Fig. 3c) revealed a statistically significant reduction in the AZT-treated mice (% of the inflammatory area in PBS-*mdx* = 0.68% vs. AZT-*mdx* = 0.35%; *p* = 0.0121; % reduction = 48,1%). Importantly, the reduction in inflammatory infiltrations was already clearly noticeable following 2 weeks of treatment. It involved significantly decreased levels of mRNAs for several inflammatory markers in both TA and GC muscles (TNFa: AZT-*mdx* vs PBS-*mdx p* = 0.038; IL12a: AZT-*mdx* vs PBS-*mdx p* = 0.017) (Additional file 4: Figure S3D).

**Fig. 4:**
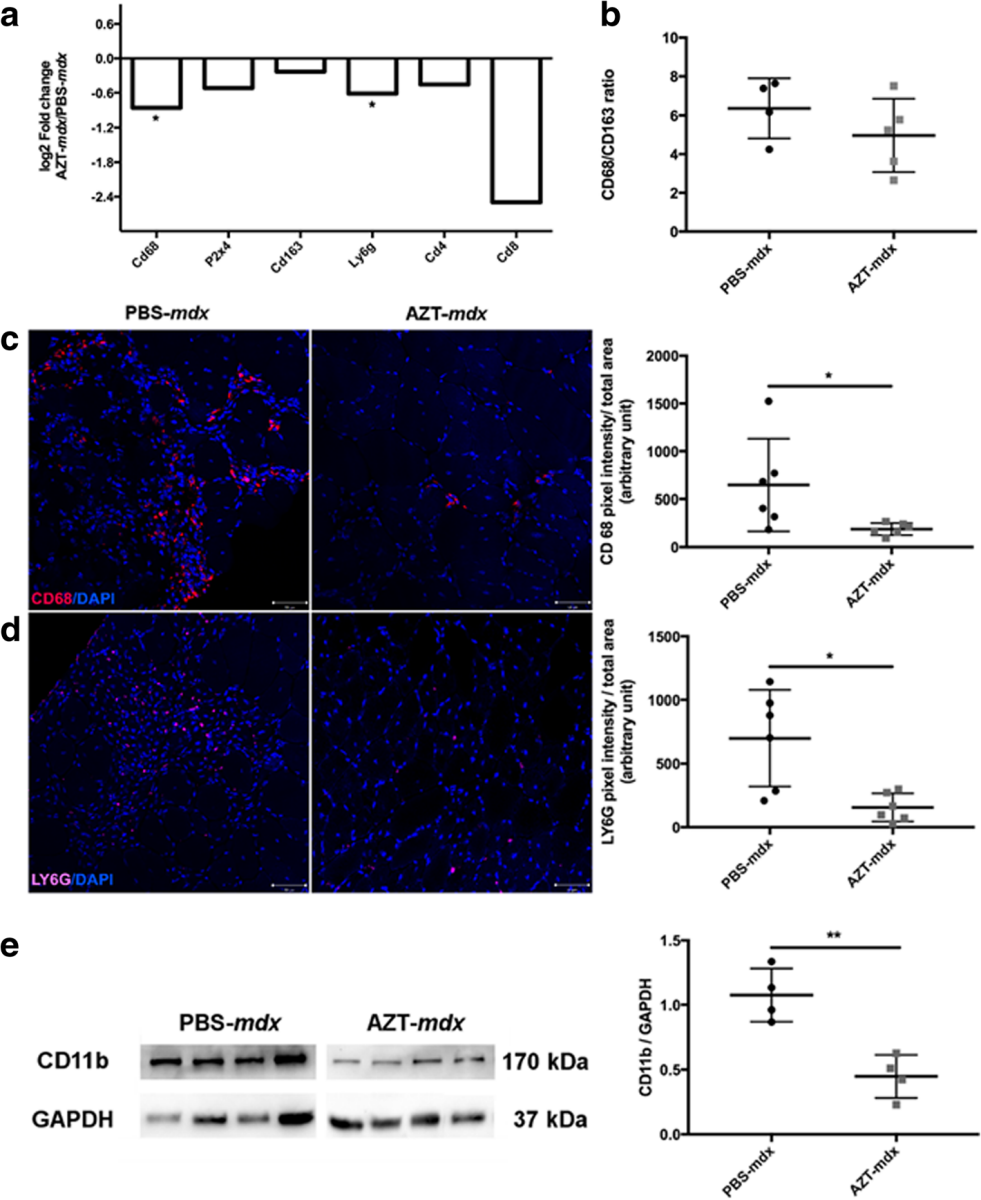
AZT modulates the immune responses in *mdx* mouse muscle. Relative expression levels of inflammatory marker RNAs in gastrocnemii following 4 week AZT treatment (**a**). The data are expressed as Log2 fold change versus the mean PBS-*mdx*. Mann Whitney test, *n* = 5–6. CD68;AZT-*mdx* vs PBS-*mdx p* = 0.026, LY6G;AZT-*mdx* vs PBS-*mdx p* = 0.03. **b** Comparison of M1 to M2 macrophages calculated as the ratio between CD68 and CD163 expression. Error bars show mean ± SD. Mann Whitney test, *n* = 4–6. Immunofluorescent analysis of inflammatory cell subpopulations in TA sections following 4 week AZT treatment. Representative images of CD68 (**c**) and LY6G staining (**d**) are shown while scatter plots demonstrate individual values of immunofluorescent signal levels in respective AZT treated and control muscles expressed in arbitrary units: pixel intensity/total area. Error bars show mean ± SD, Mann Whitney test, *n* = 6. CD68; PBS-*mdx* vs AZT-*mdx p* = 0.015, LY6G; PBS-*mdx* vs AZT-*mdx p* = 0.015. **e** Western blot of CD11b in TA muscles following 2-week treatment with GAPDH used as the protein loading control. The graph shows the individual values of the CD11b protein normalized to GAPDH, error bars show mean ± SD. Mann Whitney test, *n* = 4, *p* = 0.003. *p < 0.05, **p < 0.01

Transcript levels of genes encoding inflammatory cell markers and associated with the innate and adaptive immune responses were quantified by RT-qPCR. Among the cell markers, the CD68 transcript (which predominates in macrophages) and the Ly6G neutrophil-specific transcript levels were all significantly reduced in the AZT-treated *mdx* mice (CD68;AZT-*mdx* vs PBS-*mdx* *p* = 0.026, LY6G;AZT-*mdx* vs PBS-*mdx* *p* = 0.026) (Fig. 4a). The ratio of pro-inflammatory to pro-regenerative macrophages (M1/M2), defined by the relative CD68 to CD163 expression was not altered significantly (Fig. 4b).

Subsequent immunofluorescence analysis of these cell markers in muscle sections both confirmed and extended these findings. It revealed that already after 2 weeks of AZT treatment the numbers of macrophages, identified by P2RX4 staining, were significantly decreased (Additional file 4: Figure S3 B and C) (PBS-*mdx* = 465 AU, AZT-*mdx* = 249 AU; *p* = 0.038; % reduction = 46,32%). At 4 weeks the CD68+ cell numbers were also reduced (PBS-*mdx* = 647,8 AU, AZT-*mdx* = 189,4 AU; *p* = 0.015; % reduction = 70%, Fig. 4c).

The AZT-evoked reduction of neutrophils was also confirmed in immunofluorescent analyses of the LY6G+ population in TA muscle samples collected following 4 weeks of treatment (PBS-*mdx* = 699 AU, AZT-*mdx* = 156 AU; *p* = 0.015; % reduction = 77%, Fig. 4d). Furthermore, levels of the CD11B leukocyte marker were analyzed by immunoblot in GC muscles collected after 2 weeks of treatment. This analysis demonstrated a significant, 58% reduction in CD11B protein levels in AZT-treated animals (PBS-*mdx* = 1.075, AZT-*mdx* = 0.446; *p* = 0.003; Fig. 4e).

**Fig. 5:**
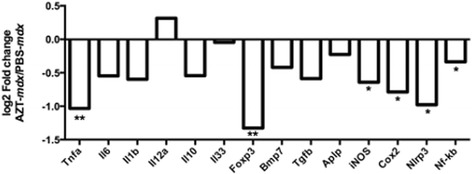
AZT treatment impacts on cytokine and inflammasome pathway gene expressions. Results of qPCR expression analyses of indicated markers in GC muscles. The data are shown as Log2 fold change versus the PBS-*mdx* and statistical analysis was performed on the relative expression values with the Mann Whitney test, *n* = 6, TNFa;AZT-*mdx* vs PBS-*mdx p* = 0.002, Foxp3;AZT-*mdx* vs PBS-*mdx p* = 0.002, iNOS;AZT-*mdx* vs PBS-*mdx p* = 0.03, COX2;AZT-*mdx* vs PBS-*mdx p* = 0.032, Nlrp3;AZT*-mdx* vs PBS-*mdx p* = 0.016, NFkb;AZT-*mdx* vs PBS-*mdx p* = 0.03.. *n* = 6; *p < 0.05 **p < 0.01

Analysis of specific cytokine transcripts in muscles (Fig. 5) revealed that TNFa levels were significantly decreased in the AZT-treated *mdx* group, consistent with the functional link between TNFa and P2RX7 (TNFa: AZT-*mdx* vs PBS-*mdx p* = 0.002). Moreover, a significant reduction of the Nlrp3 transcript levels in AZT-*mdx* muscle confirmed the Fowler et al., [22] data that AZT suppresses the Nlrp3 inflammasome by blocking P2RX7activity (Nlrp3: AZT-*mdx* vs PBS-*mdx p* = 0.03). The IL1b and IL6 expressions did not differ between treated and control groups (Fig. 5). However, expression levels of the inducible forms of cyclooxygenase COX2 and the iNOS nitric oxide synthase genes were found significantly decreased in the AZT-treated *mdx* samples, confirming that the AZT treatment affects the inflammatory milieu of *mdx* muscles (COX2: AZT-*mdx* vs PBS-*mdx p* = 0.032; iNOS: AZT-*mdx* vs PBS-*mdx p* = 0.03). This was further confirmed by the decreased expression of NF-κb in AZT-treated muscles (NF-κb: AZT-*mdx* vs PBS-*mdx p* = 0.03). This short AZT treatment did not alter the expression levels of TGFb and BMP-7 transcripts used here as previously identified markers of the early fibrotic phenotype in *mdx* muscles [58].

Concerning the adaptive immune response markers following this short-term treatment with AZT in the *mdx* mice the reduction of the CD8 and CD4 T-cell transcripts did not reach statistical significance (Fig. 4a). While ablation of *P2RX7* gene and broad purinergic inhibition with ox-ATP has previously been shown to increase the level of T regulatory (T_reg_) cells [24, 58], the AZT treatment applied here did not significantly increase the IL 12 but reduced the Foxp3 expression levels (Foxp3: AZT-*mdx* vs PBS-*mdx p* = 0.002). Expression of IL10 and IL 33 transcripts, the latter found important for muscle repair [36], was also unaltered (Fig. 5).

**Fig. 6:**
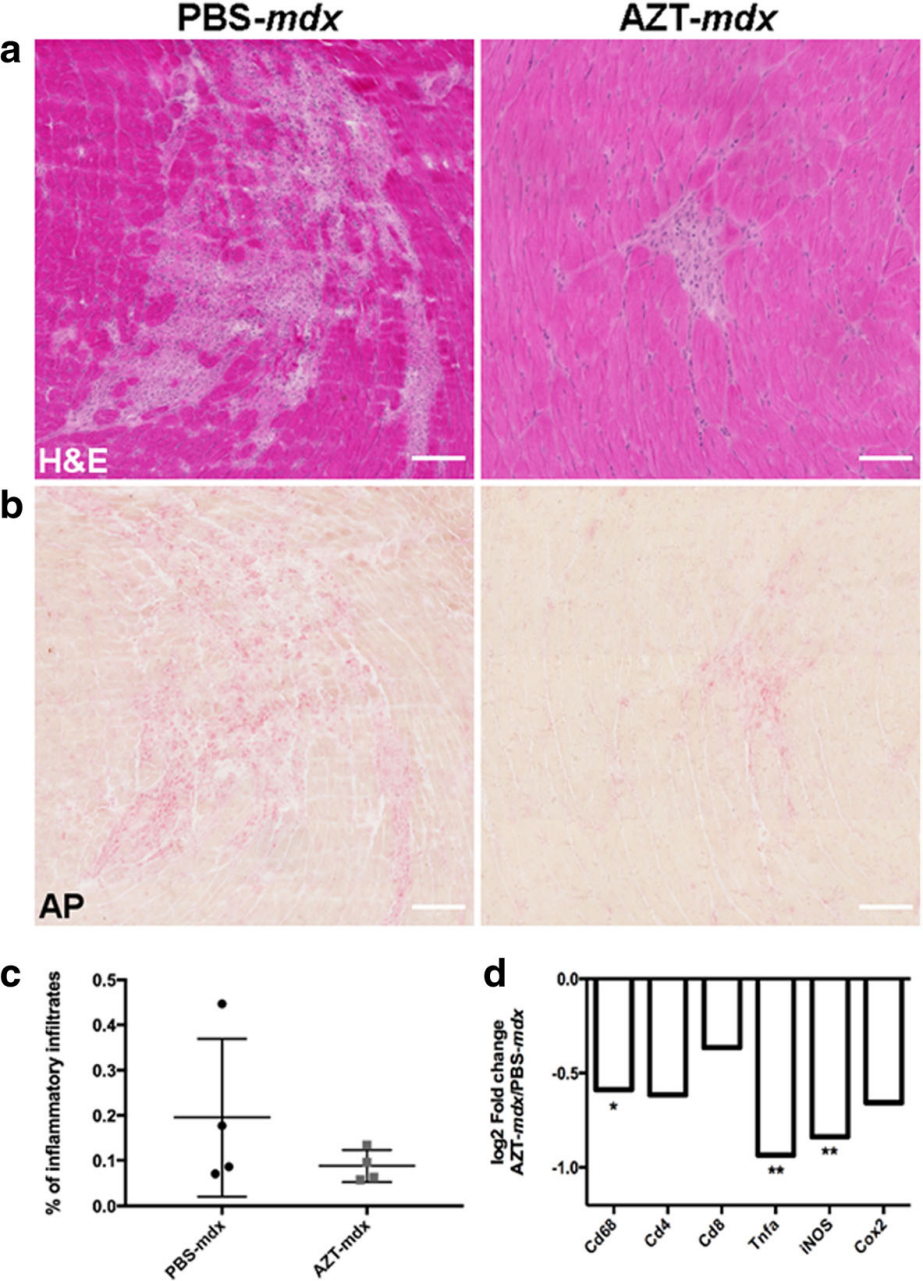
AZT treatment reduces inflammatory infiltrates and expression of inflammatory markers in *mdx* hearts. Representative images of heart muscle sections following 4-week treatment with AZT or PBS stained with H&E (**a**) and AP (**b**). **c** The AP signal indicating inflammatory cell infiltration was quantified using the Ariol image analysis system with the AP positive area calculated as % of the total cross-sectional area. Error bars show mean ± SD, Mann Whitney test, *n* = 4. **d** qPCR analysis of specified transcript expressions in heart muscles in response to AZT treatment. Scale bar = 100 μm. Mann Whitney test, *n* = 6, CD68: AZT-*mdx* vs PBS-*mdx p* = 0.029; TNFa: AZT-*mdx* vs PBS-*mdx p* = 0.008; iNOS: AZT-*mdx* vs PBS-*mdx p* = 0.009. *p < 0.05; **p < 0.01

DMD patients who survive to their third decade present with cardiomyopathy and heart failure [11, 38]. Therefore, hearts were studied to investigate the impact of AZT treatment at an early stage of disease. H&E (Fig. 6a) and AP (Fig. 6b) staining showed the presence of reactive inflammatory infiltrates in heart muscles of 8 week old *mdx* mice. The inflammation area was reduced by the AZT treatment, albeit the values did not reach significance probably due to the high variability in the controls and the small sample size (% inflammatory area PBS-*mdx* = 0,19%, AZT-*mdx* = 0,08%; % reduction = 57%, Fig. 6c). Nevertheless, RTq-PCR analysis demonstrated a highly statistically significant decrease of CD68 macrophage marker as well as TNFa and iNOS transcript levels in hearts of AZT-treated *mdx* mice (Fig. 6d), which was consistent with the findings in skeletal muscles (CD68: AZT-*mdx* vs PBS-*mdx p* = 0.029; TNFa: AZT-*mdx* vs PBS-*mdx p* = 0.008; iNOS: AZT-*mdx* vs PBS-*mdx p* = 0.009).

### The AZT treatment increases sarcolemma stability and impacts muscle strength in vivo

**Fig. 7:**
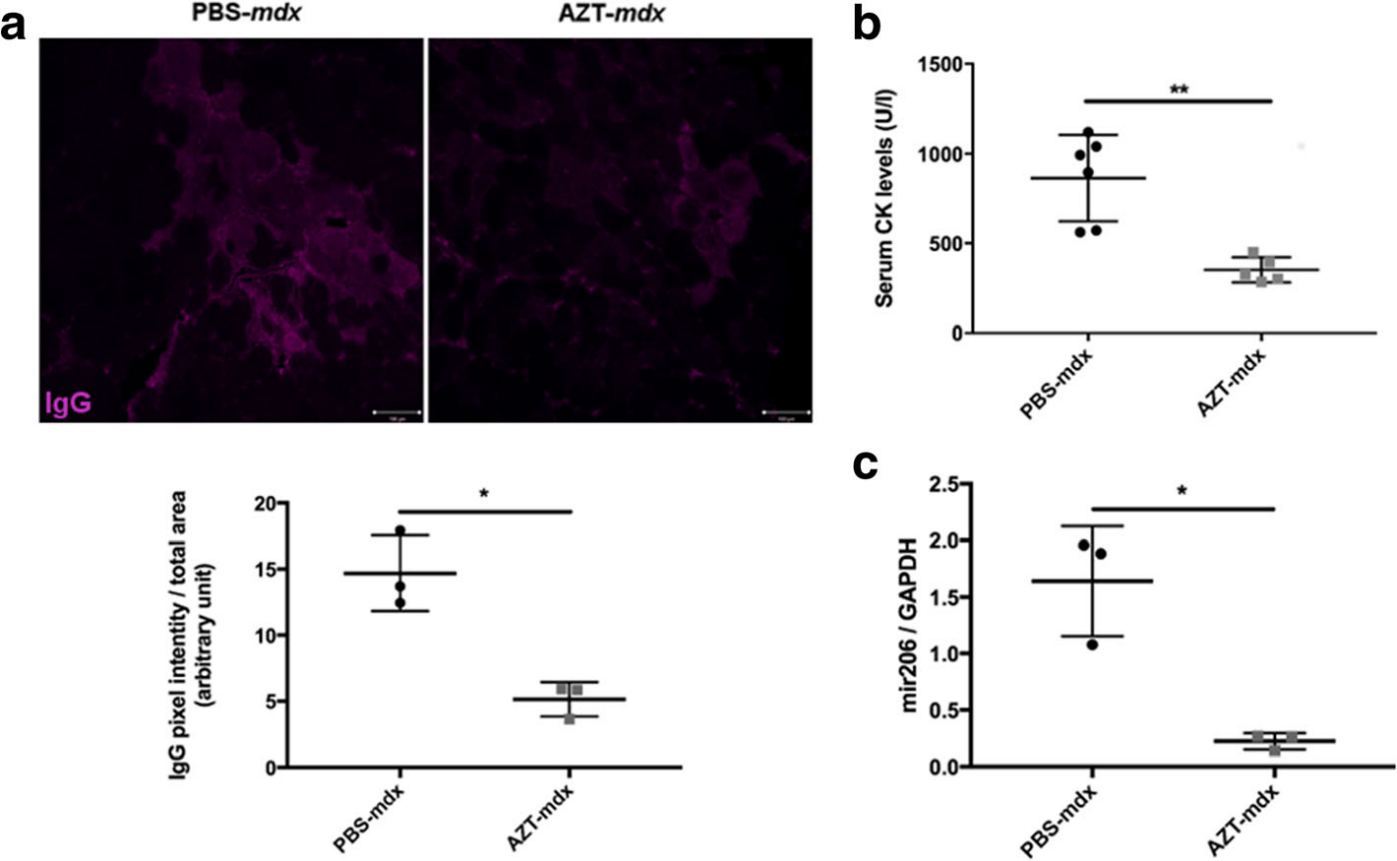
AZT treatment increases sarcolemma stability in *mdx* muscle. **a** Representative images of IgG penetration into damaged TA muscle fibers from *mdx* mice treated for 2 weeks with AZT or PBS and the graph below shows enumeration of IgG penetration represented as pixel intensity/total unit of area. Error bars show mean ± SD, Mann Whitney test, *n* = 3, *p* < 0.05 **b** Serum CK levels in AZT-treated mice (4-week treatment) compared to controls*.* Error bars show mean ± SD, Mann Whitney test, *n* = 5–6, *p* = 0.004. **c** qPCR analysis of Mir206 in serum samples from PBS or AZT treated mice following 2-week treatment. Error bars show mean ± SD. Mann Whitney test, *n* = 3, *p* = 0.029. *p < 0.05, **p < 0.01

AZT treatment resulted in significantly improved sarcolemma integrity: An analysis of membrane permeability using IgG influx into TA muscle revealed a significant reduction in IgG-positive fibers already after 2 weeks of treatment (IgG pixel intensity PBS-*mdx* = 14.7 AU, AZT-*mdx* = 5.14 AU) (Fig. 7a). After 4 weeks of AZT treatment there was a significant, 60% reduction in serum CK level (Fig. 7b), indicative of less sarcolemma damage and therefore less leakage of this intracellular muscle enzyme (serum CK levels: PBS-*mdx* = 864.37 UI/l, AZT-*mdx* = 351.72 UI/l; *p* = 0.004). The serum levels of miR-206, dystroMir, in *mdx* mice have been found to be less affected by movement compared to CK [39] and therefore this dystroMir has been proposed as a stable molecular marker of muscle damage. Here, the serum levels of Mir-206 were significantly decreased in the AZT-treated *mdx* mice after 2 weeks of treatment (Fig. 7c).

Muscle morphological parameters such as the Minimum Feret’s diameter and the number of fibers with central nucleation were evaluated in TA muscle sections from AZT- and PBS-treated *mdx* after both 2 and 4 weeks of treatment, but no statistically significant differences between the groups were observed (Additional file 5: Figure S4). Revertant fibers found in dystrophic muscle arise due to spontaneous exon skipping events, taking place in proliferating myogenic cells activated by muscle degeneration/regeneration cycles. Fewer revertant fibers have been used as an indicator that a muscle underwent fewer such cycles [47, 56]. The revertant fiber reduction was not statistically significant but no increase indicated that AZT treatment is not accelerating muscle damage (Additional file 5: Figure S4).

**Fig. 8:**
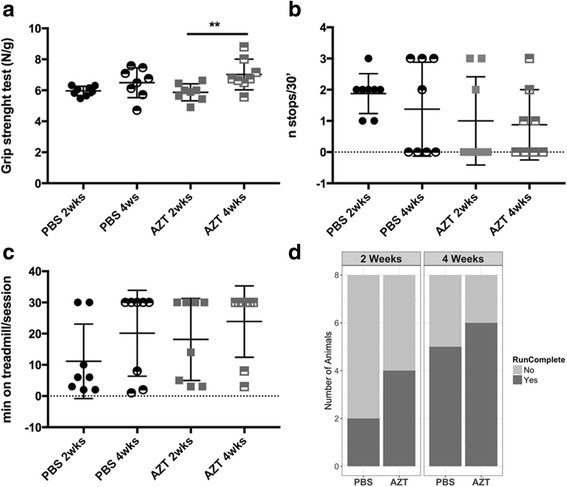
AZT improves muscle strength in *mdx* mice. **a** Forelimb grip strength measurements in control and AZT-treated *mdx* mice at the end of the second and the fourth week of treatment showed a significant improvement in the AZT group (Error bars show mean ± SD, two way-ANOVA, df = 7, *n* = 8, Bonferroni’s test, AZT:2wks vs AZT:4wks, *p* = 0.0089). **b** The number of stops per treadmill session in the analyses performed at the end of the second and fourth week of treatment. Error bars show mean ± SD, two way-ANOVA, df = 7, *n* = 8), **c** Time (in minutes) spent by individual mouse on the treadmill (Error bars show mean ± SD, two way-ANOVA, df = 7, *n* = 8) and **d** Number of PBS- or AZT-treated animals, which completed or not the 30 min treadmill session at the end of the second and fourth week of treatment (Pearson Chi-Square test, df = 2, *n* = 8). **p < 0.01

In order to establish if AZT would affect the muscle function in *mdx* mice, muscle strength and resistance were evaluated at 2 and 4 weeks of treatment using the forelimb grip strength and 30 min treadmill run tests. The single 30 min treadmill exercise session has previously been shown suitable for proof-of concept studies in adult *mdx* mice [51]. The 4 week AZT treatment improved grip strength compared to the 2 week one while no improvement with age was observed in the PBS-treated mice (Fig. 8a). Moreover, 50 and 75% of AZT-*mdx* mice in the 2 and 4-week treatment groups, respectively completed the 30 min treadmill run compared to only 25 and 62% of PBS-treated *mdx* mice, even though the result did not reach the statistical significance (Fig. 8b-d). The differences in the number of stops and the total time on the treadmill did not reach statistical significance following this short treatment regimen.

### AZT had no acute detrimental effect on mitochondria and 2-Me-AZT retains anti-P2RX7 activity

**Fig. 9:**
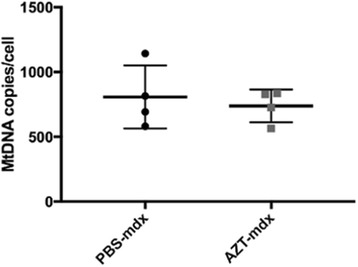
AZT treatment did not induce mitochondrial toxicity in *mdx* mice. mtDNA copy number per cell determined using qPCR (absolute quantification method) in PBS- and AZT-treated mouse muscle. Error bars show mean ± SD, Mann Whitney test, *n* = 4

It is known that NRTIs, including AZT, can affect mtDNA-specific polymerase (polymerase-gamma) activity, which can lead to deficiencies in mtDNA maintenance and transcription, and consequently mitochondrial abnormalities, especially in tissues requiring high energy, such as muscles. Notably, 100 mg/kg/day i.p. used here is somewhat higher than the equivalent human i.v. therapeutic dose (e.g. 8.1 vs. 6 mg/kg). Therefore, we analyzed the number of mtDNA copies in TA muscles from animals that received AZT treatment for 4 weeks. No differences in the amount of mtDNA copies were observed (Fig. 9), indicating that this AZT treatment had no immediate detrimental effect on the mitochondrial replication.

Nevertheless, appreciating the possibility of side effects caused by a chronic AZT treatment, we performed a preliminary study with 2-Me-AZT, a compound shown not to affect DNA polymerases [22, 44] but to retain its anti-P2RX7 activity (Fig. 2b). Following a 4-week treatment, RT-qPCR analysis of key inflammatory cell markers (CD68, PRX4, LY6G, Foxp3, TNFa) showed that this compound triggers a significant reduction of these inflammatory parameters in the treated *mdx* muscle (CD68: AZT-*mdx* vs PBS-*mdx p* = 0.03; LY6G: AZT-*mdx* vs PBS-*mdx p* = 0.026; TNFa: AZT-*mdx* vs PBS-*mdx p* = 0.002) (Additional file 6: Figure S5). This result confirms the observations of Fowler et al., [22] and indicates that this drug and/or derivatives of other NRTIs could be developed as safer alternatives for treatment of DMD and other diseases involving over-activation of P2RX7.

## Discussion

Loss of dystrophin disrupts various downstream processes. Recent studies demonstrated that such abnormalities can be good targets for therapeutic interventions [24, 25, 35, 46, 58, 67, 68, 70, 72]. Unlike exon skipping or Ataluren, aimed at restoration of dystrophin, these treatments are not constrained by the causative DMD mutation. Therefore, all DMD patients rather than just a small subpopulation might benefit from these therapies. Moreover, these treatments may be effective in alleviating multiple abnormalities: muscle cell damage, inflammation, cognitive impairment and bone defects, which all make a substantial contribution to the clinical picture of DMD.

We demonstrated here that P2RX7 inhibition using a short treatment with AZT at the peak of disease severity in *mdx* mice attenuated the phenotype without any detectable side effects. The recovery was evident in key pathological parameters in treated leg and heart muscles such as reduced serum CK levels, decreased inflammatory markers and increased muscle strength in vivo.

The interest in P2RX7 as a therapeutic target in DMD results from the recent discovery that dystrophinopathy causes functional alterations of this purinoceptor and that the genetic ablation and pharmacological inhibition of P2RX7 in *mdx* mice produced significant improvements in key functional and molecular disease parameters [24, 58, 74]. In addition to the alleviation of muscle disease and decreased inflammation, reduced non-muscle symptoms (CNS and bones) were also evident [41, 58].

The wide therapeutic impact of P2RX7 inhibition reflects convergence of P2RX7 on multiple pathological mechanisms of DMD: P2RX7 inhibition reduces dystrophic muscle cell death directly [54, 73, 75], prevents the MMP-evoked damage [74] and also inhibits chronic inflammation [17, 24]. The association of P2RX7 with inflammation and immunity is long established. P2RX7 is involved in a range of responses including cytokine release, lymphocyte proliferation, intracellular pathogen killing, stimulation of gut mucosal immunity and even pain perception [15, 17, 43]. Not surprisingly, P2RX7 activation has been linked with a number of human diseases with an inflammatory component [22, 64, 66, 69, 71]. However, there is increasing evidence that P2RX7 also plays a pivotal role in central nervous system pathologies and in psychiatric disorders, where the link with inflammation is not always clear [61]. Indeed, the P2RX7 expression can be modulated in non-immune cells and it changes in disease states. For example, in addition to the aforementioned increased P2RX7 expression and function in dystrophic muscle [73–75], over-expression of this purinoceptor is evident in various human cancer cells [1, 16, 18].

Given the range of diseases involving this receptor, P2RX7 has attracted a lot of attention as a therapeutic target and specific antagonists have been developed. However, none of these are currently available as a drug. Moreover, each would need to undergo a lengthy and expensive evaluation process prior to use in a pediatric population such as for DMD.

Therefore, we studied AZT’s potential to ameliorate DMD symptoms by blocking the P2RX7-evoked effects. We established, through molecular modelling, that both AZT and its methylated derivative could potentially bind with high affinity to the same allosteric site found to be occupied by the canonical antagonists. This site is distinct from the ATP-binding pocket and can accommodate structurally-similar but diverse molecules, mostly through hydrophobic interactions. Allosteric binding of these compounds prevents narrowing of the drug-binding pocket, a process crucial for opening the P2RX7 channel. Interestingly, the equivalent pocket in other P2X receptors is too narrow to accommodate the P2RX7 antagonist and therefore the size difference confers binding specificity of P2RX7 inhibitors [31]. In vitro assays confirmed that AZT could prevent the channel and the large pore opening in dystrophic myoblasts.

Using the most widely exploited animal model of DMD, we provided evidence that even a short (2 weeks) treatment with AZT causes P2RX7 inhibition in vivo. This was evidenced by the decreased TNFa levels, the production of which is P2RX7-dependent [10]. Given that pharmacological interference with TNFa has been shown to reduce DMD pathology [25], reduction of this inflammatory mediator is also clinically important. Indeed, AZT therapy improved muscle functions and sarcolemma stability measured by reduced CK and MiR206 serum levels and inhibited influx of IgG into muscle fibers. The most evident beneficial effect of AZT was a significant dampening of inflammation. AZT decreased the inflammatory *milieu* of dystrophic muscles chiefly by influencing the innate response. The inflammatory cell repertoire analysis in treated *mdx* skeletal and cardiac muscles showed that the treatment led to a reduction of markers for neutrophil and macrophage populations, which was noticeable just after 2 weeks of treatment and enhanced at 4 weeks.

Significantly reduced neutrophil marker Ly6G levels were found in AZT-treated samples, which corresponded with our previous findings in *mdx*/P2RX7 double mutant mice [58]. Hodgetts et al. [29] showed neutrophil depletion to be very effective in reducing myofibers necrosis in young *mdx* mice. Neutrophil-mediated skeletal muscle injury seems dependent on the free radical production and is tightly associated with the cytokines profile [29, 53]. Interestingly, in AZT-treated *mdx* we found remarkably reduced transcript levels of TNFa and of the inducible forms of COX and NOS (COX2 and iNOS). AZT may trigger neutrophil down-regulation and thus reduce inflammation by directly inhibiting P2RX7 in these cells: A recent study showed that both human and murine neutrophils express functional P2RX7, activation of which leads to NLRP3 inflammasome activation and IL-1b secretion [32]. Moreover, P2RX7 ablation has a widespread impact on the inflammatory cell migration, including neutrophils [33] and this might also explain the reduced neutrophil load in dystrophic muscles.

AZT treatment did not significantly reduce the levels of CD4+ or CD8+ T-cells and this short term treatment did not unequivocally affect expression levels of Foxp3 and IL12a, markers of the T_reg_ cells. Given that our previous studies demonstrating this enhancement of muscle T_reg_ involved the genetic ablation of P2RX7 or global purinergic inhibition [24, 58], further experiments are needed to establish whether sustained AZT treatment or different dosing regimen would result in a shift in T-cell responses.

The reduction of inflammatory cell infiltrations and inflammatory markers expression in cardiac muscles following AZT treatment was also in agreement with our previous data in mice with blocked P2RX7 receptors [24, 58]. This finding is of clinical importance because heart failure becomes the most common cause of death in DMD patients surviving longer due to advances in general care [11].

Taken together, these results demonstrate that AZT, targeting P2RX7 functions in both muscle and inflammatory cells, reduces the production of pro-inflammatory mediators thus leading to reduced inflammation with fewer lymphocytes, neutrophils and macrophages within *mdx* muscles. There is also reduced muscle membrane damage and slightly improved muscle strength in AZT-treated dystrophic mice. In experimental paradigms not inducing dystrophin exon skipping, such as one described here, fewer revertant fibers have been used as an indicator that a muscle underwent fewer degeneration/regeneration cycles [47, 56]. Not unexpectedly, given the short treatment period, no significant reduction in the revertant fiber count was found. However, this result indicated that AZT has no negative effect as any disease acceleration should be quickly reflected in muscles’ degeneration/regeneration history.

AZT is a mainstay in the prevention of mother-to-child HIV transmission. A recent extensive survey on the safety of AZT in the pediatric population in the EU and Thailand has shown AZT to be a well-tolerated drug with few side effects [65], thus confirming earlier findings in the pediatric population [50] High NRTI dosage can interfere with the eukaryotic mtDNA-specific polymerase. However, these side-effects occur in long term treatment regimens and are reversible: Elimination half-life following i.v. administration is ~ 1.1 h [6, 23]. Notably, 100 mg/kg/day i.p*.* used here is higher than the equivalent human i.v. dose (8.1 vs. 6 mg/day). While this treatment had no detrimental effect on mitochondria, which are typically the most sensitive organelles in NRTIs therapy, a longer-term and different dosing regimen would help correlating the therapeutic impact and potential side-effects of AZT fully. Appreciating the possibility of side effects of a chronic AZT treatment, we also tested its methyl derivative (2-Me-AZT) not affecting DNA polymerases [22, 44] but found by us to bind the same allosteric site and to retain anti-P2RX7 activity. Following a 4-week treatment, we demonstrated that 2-Me-AZT caused a significant reduction of inflammatory parameters akin to the effects of AZT.

## Conclusions

In conclusion, AZT emerges as a P2RX7 inhibitor effective in reducing harmful inflammation and potential to improve muscle function in dystrophic *mdx* mice. Given extensive safety and pharmacokinetic data available following decades of AZT use in humans, also in children and neonates, this drug should be considered for trials in DMD. Unlike other experimental drugs, it could be used from the very early stage of this debilitating and ultimately lethal disease, where any treatment is likely to be most effective.

## Additional files

Additional file 1: **Figure S1.** Human, giant panda and mouse P2RX7 peptide sequence alignments. (PDF 779 kb)

Additional file 2: **Table S1.** Binding Energies for AZT at all of the sites in the 5U1V identified by the site finder in MOE. (DOCX 16 kb)

Additional file 3: **Figure S2.** Average weight gain of mice following AZT treatment. (TIFF 756 kb)

Additional file 4: **Figure S3.** Impact of the 2-week AZT treatment on key pathological parameters. (TIFF 6394 kb)

Additional file 5: **Figure S4.** Muscle fiber analysis following AZT administration in vivo. (TIFF 3875 kb)

Additional file 6: **Figure S5.** 2-Me-AZT treatment impact on the expression of selected inflammatory genes. (TIFF 900 kb)
