# Supplementary material for: Zidovudine ameliorates pathology in the mouse model of Duchenne muscular dystrophy via P2RX7 purinoceptor antagonism
Source: Acta Neuropathol Commun. 2018 Apr 11;6:27. doi: 10.1186/s40478-018-0530-4 (PMC5896059; doi:10.1186/s40478-018-0530-4)
Supplement: Supplementary file 1 — Figure S1. Human, giant panda and mouse P2RX7 peptide sequence alignments. (PDF 779 kb) [file 40478_2018_530_MOESM1_ESM.pdf]

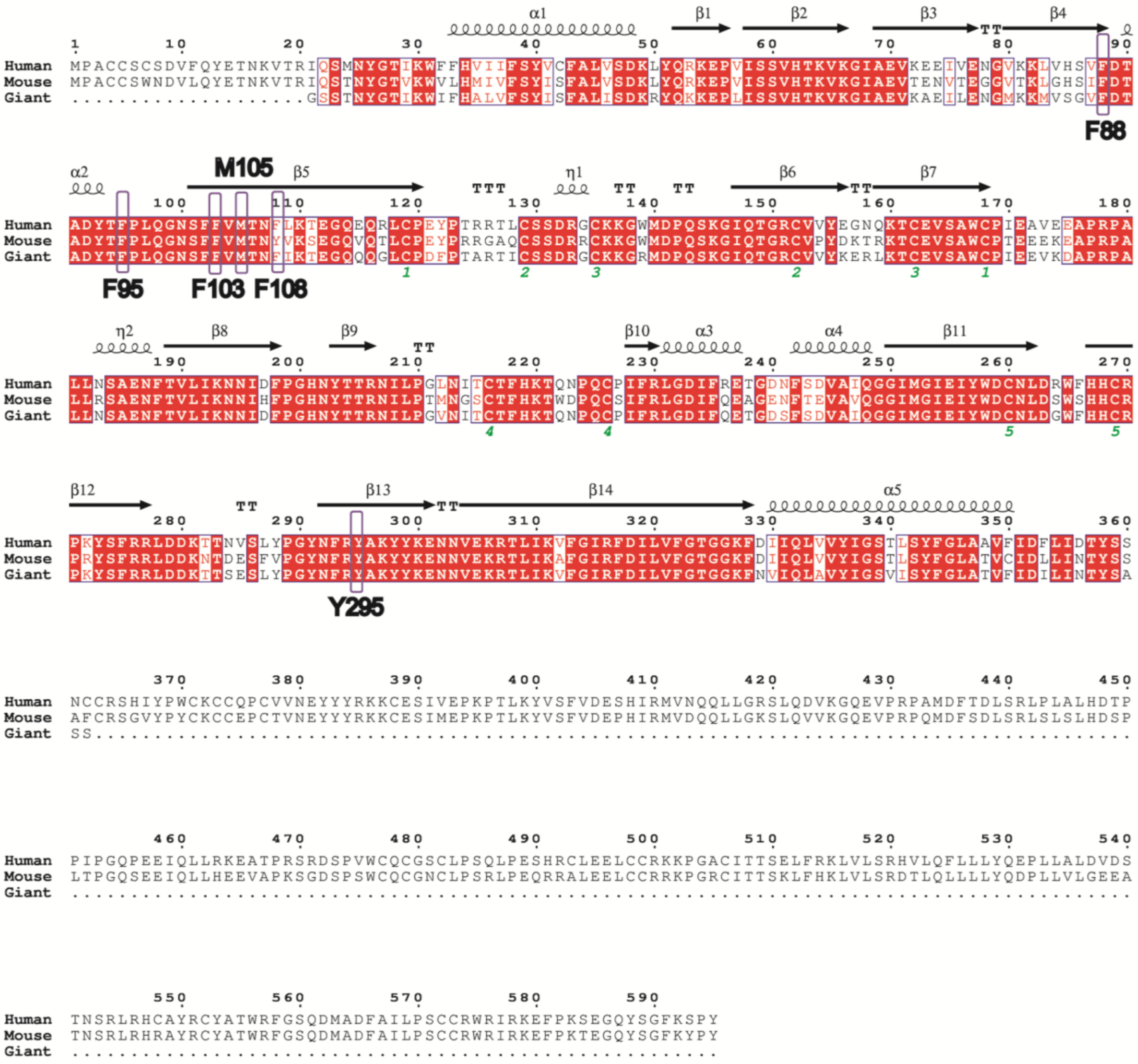

Figure S1

P2RX7 peptide sequence alignments.

Human, giant panda and mouse P2RX7 peptide sequences (UniProt) were aligned and revealed 77% and 81% of residues to be conserved in panda compared to mouse and human, respectively (highlighted in red). The key residues involved in binding the specific P2RX7 antagonists are also conserved (highlighted by purple boxes). Secondary structural elements present in the giant panda model are also depicted.
