# Supplementary material for: Zidovudine ameliorates pathology in the mouse model of Duchenne muscular dystrophy via P2RX7 purinoceptor antagonism
Source: Acta Neuropathol Commun. 2018 Apr 11;6:27. doi: 10.1186/s40478-018-0530-4 (PMC5896059; doi:10.1186/s40478-018-0530-4)
Supplement: Supplementary file 2 — Table S1. Binding Energies for AZT at all of the sites in the 5U1V identified by the site finder in MOE. (DOCX 16 kb) [file 40478_2018_530_MOESM2_ESM.docx]

Table S1: Binding Energies for AZT at all of the sites in the 5U1V identified by the site finder in MOE

| Site | Residues at Search Site | E_Refine / kJ mol^-1^ | E_Score / kJ mol^-1^ |
| --- | --- | --- | --- |
| 1 | PHE88 ALA91 ASP92 TYR93 THR94 PHE95 PRO96 PHE103 MET105 PHE108 LYS110 GLU112 GLY113 TRP167 PHE293 ARG294 TYR295 ALA296 LYS297 TYR298 TYR299 LYS300 GLU301 GLU305 THR308 ILE310 VAL312)2:(PHE88 ALA91 ASP92 TYR93 THR94 PHE95 PRO96 PHE103 MET105 PHE108 LYS110 GLU112 GLY113 TRP167 PHE293 ARG294 TYR295 ALA296 LYS297 TYR298 TYR299 LYS300 GLU301 GLU305 THR308 ILE310 VAL312)3:(PHE88 ALA91 ASP92 TYR93 THR94 PHE95 PRO96 PHE103 MET105 PHE108 LYS110 GLU112 GLY113 TRP167 PHE293 ARG294 TYR295 ALA296 LYS297 TYR298 TYR299 LYS300 GLU301 GLU305 THR308 ILE310 VAL312 | -89.32 | -27.57 |
| 2 | ILE109 ASP121 PHE122 THR124 ARG126 THR127 PRO142 GLN143 SER144 LYS145 GLY146 ILE170 GLU171 GLU172 VAL173 LYS174 ASP175 ALA176 GLN248 GLU285 SER286 LEU287 TYR288 PRO289 GLY290 TYR291 ASN292 LYS311 PHE313)3:(LYS64 LYS66 THR189 LEU191 ILE214 THR215 CYS216 THR217 ILE228 | -74.14 | -21.34 |
| 3 (5) | ASP244 VAL245 ILE247 GLN248 GLY249 GLY250 ILE251 ARG277 LEU278 ASP279 ASP280 LYS281 THR282 THR283 LEU287 TYR288 PRO289 GLY290 ARG316)2:(SER60 VAL61 HIS62 LYS193 ASN194 ASN195 ARG206 ASN207 ILE208 | -67.20 | -20.54 |
| 4 (8) | LYS145 TYR291 ASN292 PHE293 ARG294 LYS311 ARG316)2:(HIS62 THR63 LYS64 VAL65 LYS66 THR90 ALA91 THR94 PHE95 PRO96 LEU97 PHE102 PHE103 | +80.04 | -22.68 |
| 5 (10) | LYS53 LYS54 GLU55 PRO56 LEU57 ILE58 PHE198 PRO199 GLY200)2:(LYS54 GLU55 PRO56 LEU57 TYR257 TRP258 ASP259 VAL321 PHE322 GLY323 THR324 | -67.95 | -22.93 |
| 6 (13) | THR63 LYS64 VAL65 LYS66 THR90 ALA91 THR94 PHE95 PRO96 LEU97 PHE102 PHE103)2:(LYS145 ASN292 PHE293 ARG294 LYS311 | +61.80 | -22.72 |
| 7 (14) | ASP121 ARG126 THR127 ILE128 CYS129 GLY134 CYS135 LYS136 ARG139 MET140 ASP141 SER144 GLY146 ILE147 GLN148 THR161 CYS162 VAL164 | -54.31 | -18.03 |
| 8 (17) | LYS82 MET83 VAL84 SER85 GLY86 VAL87 PHE88 PHE108 LYS110 GLU112)2:(GLN116 TRP167 GLU305 | -63.05 | -22.30 |
| 9 (20) | GLN98 GLY99 ASN100 SER101 LEU278 ARG316 ASP318 ILE319 LEU320 VAL321)3:(SER59 SER60 VAL61 LEU97 GLN98 GLY99 ASN100 | -63.30 | -23.76 |
| 10 (23) | GLU255 TYR257 LYS272 SER274 PHE275 ARG276 PHE322)3:(ASP197 PHE198 PRO199 ASN202 TYR203 THR204 | -61.38 | -19.66 |
| 11 (25) | 1:(ASP197 PHE198 PRO199 ASN202 TYR203 THR204)2:(GLU255 TYR257 LYS272 SER274 PHE275 ARG276 PHE322) | -60.38 | -19.71 |
| 12 (26) | 3:(GLN116 GLY117 LEU118 SER165 ALA166)2:(GLU70 VAL71 LYS72 SER85 GLY86 VAL87 | -51.67 | -18.41 |
| 13 (29) | 3:(SER342 GLY345 LEU346 VAL349)2:(ILE341 SER342 PHE344 GLY345) | -67.03 | -21.30 |
| 14 (31) | 1:(ASP48 LYS49 ARG50 GLN52 LYS53 LYS54 ASN329 ILE331 GLN332 VAL335 TYR336 SER339)3:(VAL335)2:(ASP48 ARG50 LYS54) | -69.37 | -20.88 |
| 15 (32) | 3:(ILE75 GLU77 LYS82 VAL84 PHE108 ILE109 LYS110 LYS174 PRO177 LEU181) | -51.00 | -17.87 |
| 16 (35) | 1:(PHE95 PRO96 GLN98 TYR295)3:(PHE95 PRO96 GLN98 TYR295)2:(PHE95 PRO96 GLN98 TYR295) | -69.75 | -25.52 |
| 17 (36) | 3:(LYS49 GLN52 LYS54 ASP259 CYS260 ASN261 LEU262 HID268 THR324 GLY325 GLY326) | -59.16 | -18.87 |
| 18 (38) | 1:(SER342 GLY345 LEU346 VAL349)3:(ILE341 SER342 PHE344 GLY345 VAL349)2:(SER342 GLY345 VAL349) | -67.32 | -21.13 |
| 19 (39) | 1:(VAL65 LYS66 GLY67 ILE68 THR90)2:(MET140 ASP141 PRO142 GLN143 SER144 LYS145) | -55.15 | -19.92 |
| 20 (40) | 3:(MET140 ASP141 PRO142 GLN143 SER144 LYS145)2:(VAL65 LYS66 GLY67 ILE68 ASP89 THR90 TYR93) | -52.68 | -20.54 |
| 21 (42) | 2:(LYS49 GLN52 LYS54 ASP259 CYS260 ASN261 LEU262 THR324 GLY325 GLY326) | -58.53 | -19.00 |
| 22 (43) | 1:(GLU74 ILE75 LEU76 LYS81 ILE109 LYS174 ASP175 ALA176 PRO177 ARG178 PRO179) | -60.21 | -20.04 |
| 23 (46) | 2:(LEU76 GLU77 ASN78 LYS82 ILE109 LYS110 THR111 GLU112 PRO169 ILE170 GLU171 GLU172 LYS174) | -52.80 | -19.62 |
| 24 (49) | 1:(LYS53 GLY200 HIS201 LYS327 PHE328 ASN329) | -51.21 | -18.08 |
| 25 (52) | 3:(THR238 ASP240 ARG276 ARG277 LEU278 LYS281)2:(ASN195 THR204 ARG206) | -61.25 | -19.41 |
| 26 (55) | 2:(PHE218 HIS219 LYS220 PRO224 GLN225 PRO271 LYS272 TYR273) | -49.12 | -18.87 |
| 27 (58) | 1:(LEU57 ILE58 SER59 ASN100)2:(LEU320 VAL321 PHE322) | -63.43 | -21.59 |
| 28 (61) | 1:(ASP48 ARG50 LYS54)3:(ASP48 LYS49 ARG50 GLN52 LYS53 LYS54 ASN329 ILE331 GLN332) | -69.58 | -20.79 |
| 29 (63) | 2:(ILE46 TYR51 TRP265) | -60.67 | -18.83 |
