# Supplementary figures and images for: Zidovudine ameliorates pathology in the mouse model of Duchenne muscular dystrophy via P2RX7 purinoceptor antagonism
Source: Acta Neuropathol Commun. 2018 Apr 11;6:27. doi: 10.1186/s40478-018-0530-4 (PMC5896059; doi:10.1186/s40478-018-0530-4)

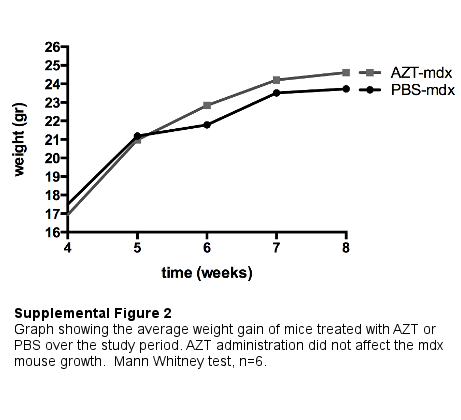

Supplement: Supplementary file 3 — Figure S2. Average weight gain of mice following AZT treatment. (TIFF 756 kb) [file 40478_2018_530_MOESM3_ESM.tiff]

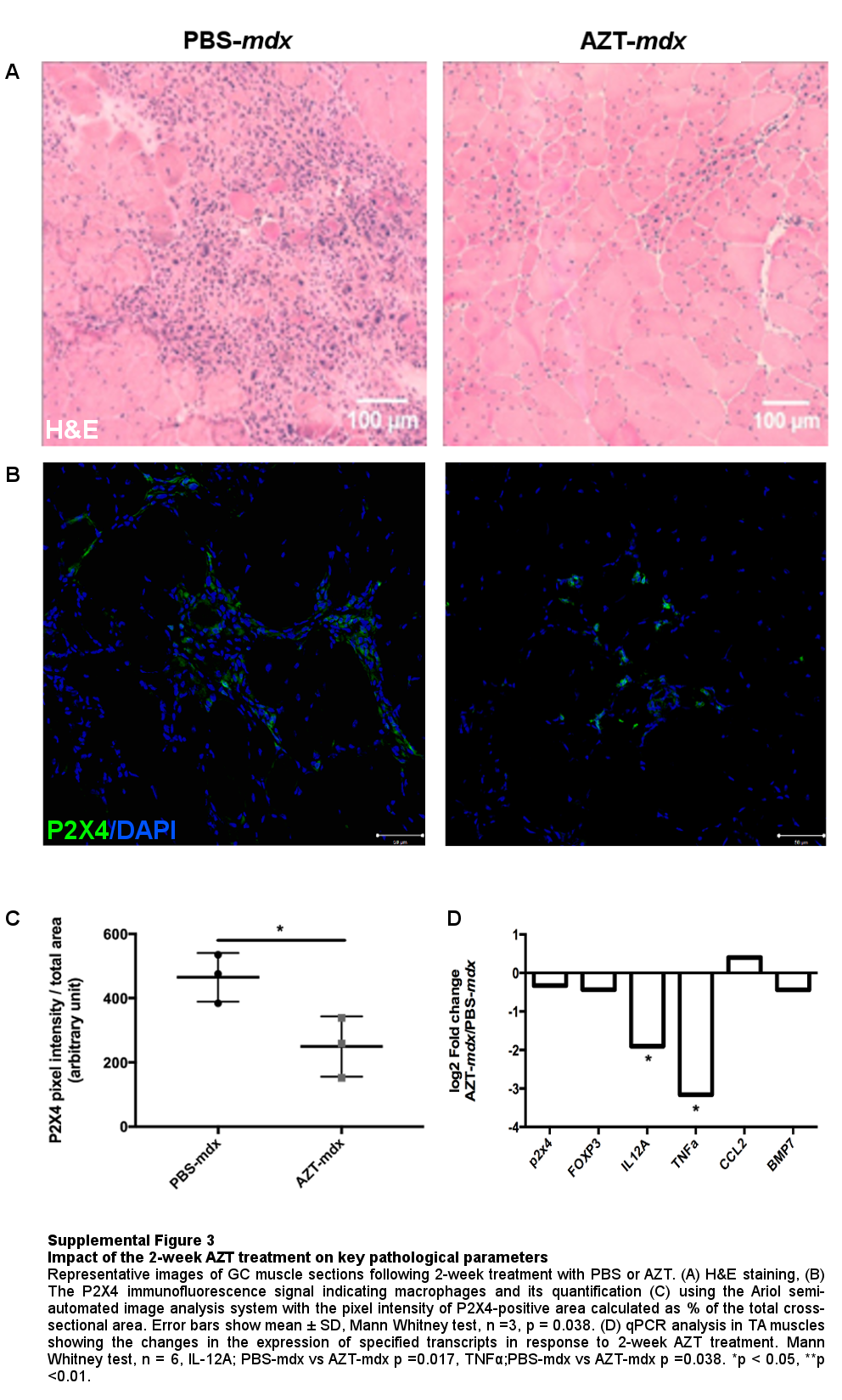

Supplement: Supplementary file 4 — Figure S3. Impact of the 2-week AZT treatment on key pathological parameters. (TIFF 6394 kb) [file 40478_2018_530_MOESM4_ESM.tiff]

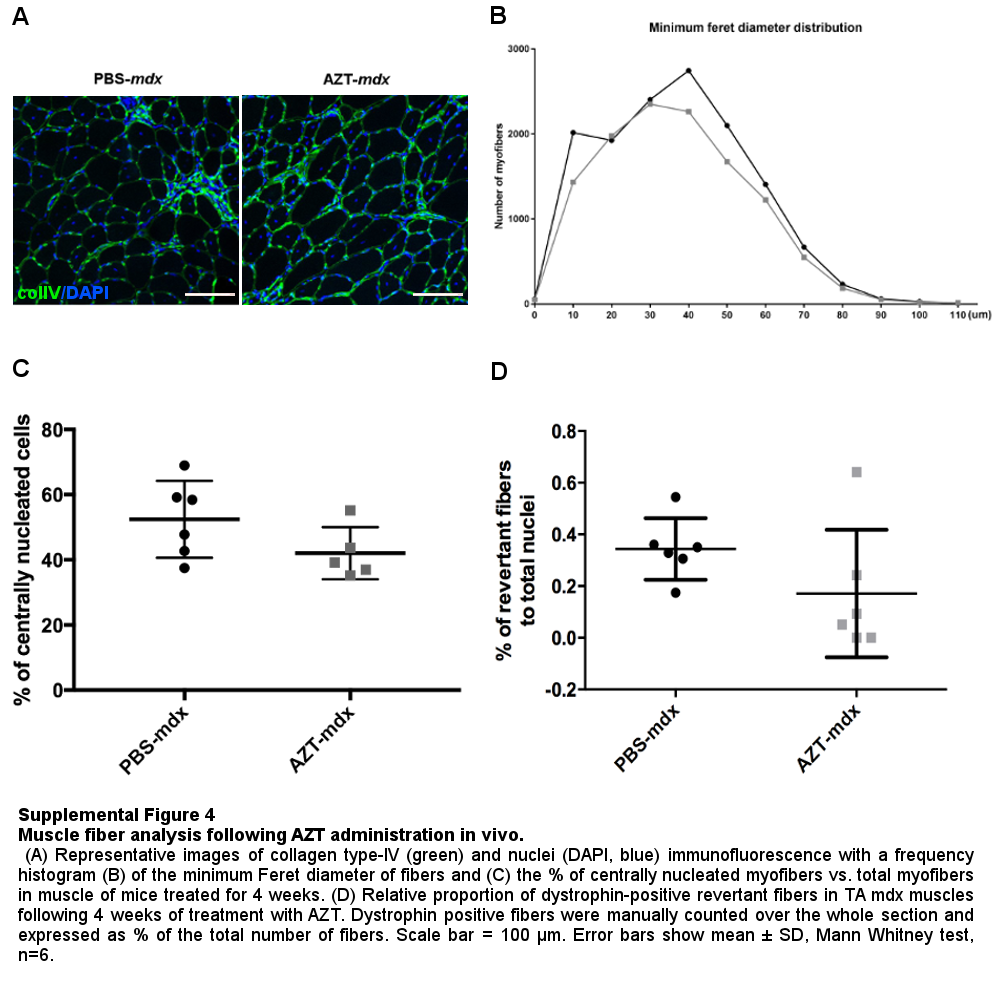

Supplement: Supplementary file 5 — Figure S4. Muscle fiber analysis following AZT administration in vivo. (TIFF 3875 kb) [file 40478_2018_530_MOESM5_ESM.tiff]

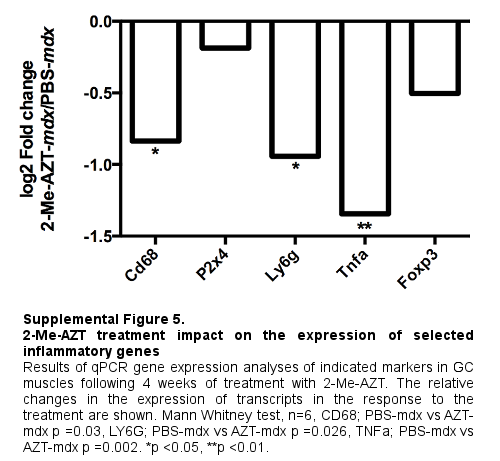

Supplement: Supplementary file 6 — Figure S5. 2-Me-AZT treatment impact on the expression of selected inflammatory genes. (TIFF 900 kb) [file 40478_2018_530_MOESM6_ESM.tiff]
